# The distribution, diversity, and importance of 16S rRNA gene introns in the order Thermoproteales

**DOI:** 10.1186/s13062-015-0065-6

**Published:** 2015-07-09

**Authors:** Zackary J. Jay, William P. Inskeep

**Affiliations:** Thermal Biology Institute and Department of Land Resources and Environmental Sciences, Montana State University, Bozeman, MT USA

**Keywords:** *Archaea*, Thermoproteales, Introns, 16S rRNA, Geothermal, Metagenomics

## Abstract

**Background:**

Intron sequences are common in 16S rRNA genes of specific thermophilic lineages of *Archaea*, specifically the Thermoproteales (phylum Crenarchaeota). Environmental sequencing (16S rRNA gene and metagenome) from geothermal habitats in Yellowstone National Park (YNP) has expanded the available datasets for investigating 16S rRNA gene introns. The objectives of this study were to characterize and curate archaeal 16S rRNA gene introns from high-temperature habitats, evaluate the conservation and distribution of archaeal 16S rRNA introns in geothermal systems, and determine which “universal” archaeal 16S rRNA gene primers are impacted by the presence of intron sequences.

**Results:**

Several new introns were identified and their insertion loci were constrained to thirteen locations across the 16S rRNA gene. Many of these introns encode homing endonucleases, although some introns were short or partial sequences. *Pyrobaculum*, *Thermoproteus*, and *Caldivirga* 16S rRNA genes contained the most abundant and diverse intron sequences. Phylogenetic analysis of introns revealed that sequences within the same locus are distributed biogeographically. The most diverse set of introns were observed in a high-temperature, circumneutral (pH 6) sulfur sediment environment, which also contained the greatest diversity of different Thermoproteales phylotypes.

**Conclusions:**

The widespread presence of introns in the Thermoproteales indicates a high probability of misalignments using different “universal” 16S rRNA primers employed in environmental microbial community analysis.

**Reviewers:**

This article was reviewed by Dr. Eugene Koonin and Dr. W. Ford Doolittle.

**Electronic supplementary material:**

The online version of this article (doi:10.1186/s13062-015-0065-6) contains supplementary material, which is available to authorized users.

## Background

The 16S rRNA is the central structural component of the bacterial and archaeal 30S ribosomal subunit and is required for the initiation of protein synthesis and the stabilization of correct codon-anticodon pairing in the A site of the ribosome during mRNA translation [1]. Due to the functional constancy and highly conserved nature of the 16S rRNA gene, it has been an important phylogenetic marker, and was used to define the three domains of Life [2–4]. Several lineages within the domain *Archaea* contain 16S rRNA gene introns, which are mobile genetic elements that do not appear to impact the host’s growth or metabolism. 16S rRNA gene introns have been identified in two genera of the order Desulfurococcales (*Aeropyrum* and *Staphylothermus*) [5–10] and four genera in the order Thermoproteales: *Pyrobaculum* (11 spp.), *Thermoproteus* (2 spp.), *Caldivirga* (1 sp.), and *Vulcanisaeta* (1 sp.) [10–19]. The only other archaea to contain 16S rRNA gene introns are the Aigarchaeota (i.e., *Caldiarchaeum subterraneum*) [20]. The orders Desulfurococcales and Thermoproteales also contain 23S rRNA and tRNA gene introns; however, the limited number of genomes in these orders prevents a robust analysis of the diversity and distribution of introns in these genes.

Currently, the Thermoproteales exhibit the most numerous and diverse rRNA and tRNA introns in the domain *Archaea* [21–23]. Although the life cycle of tRNA and rRNA intron sequences has been well characterized, little is known regarding the transmission and evolution of introns [23, 24]. A distinguishing characteristic of all archaeal rRNA and tRNA introns is the bulge-helix-bulge motif formed at the intron insertion site [25, 26]. This core motif is recognized by the tRNA splicing endoribonuclease, which is responsible for post-transcriptional excision of RNA introns [27–30] followed by ligation via the tRNA splicing ligase (RtcB; [31]) during the maturation process. Many rRNA introns encode homing endonuclease proteins with either one or two copies of the characteristic LAGLI-DADG motif [24, 32]. The two forms of the enzyme (a homodimer, which consists of two subunits each containing one motif copy, and a monomer, which is a single subunit containing two motif copies) each recognize long stretches (15 - 30 bp) of intron^–^ loci and catalyze a double-strand break where the intron sequence is inserted via recombination and repair mechanisms [10, 33]. Frequent horizontal transmission within a population is required for intron persistence, while the remnant sequence is subjected to decreased selection pressure [10, 34]. A functional homing endonuclease gene or a mRNA transcript has been suggested for intercellular intron migration [25, 35].

Direct sequencing of 16S rRNA genes and/or metagenomes from environmental samples has become a standard and convenient method of assessing microbial population abundance, structure, and function in microbial communities (e.g. [36, 37]). Publically available 16S rRNA gene sequences from high-temperature geothermal environments in Yellowstone National Park (YNP) have increased dramatically, which provides a large dataset to determine the diversity and distribution of introns in (hyper)thermophilic archaeal communities. Consequently, the objectives of this study were to (i) characterize and curate all archaeal 16S rRNA gene introns found within currently available genomes and environmental sequence databases, (ii) perform a phylogenetic analysis to evaluate the conservation and distribution of archaeal 16S rRNA introns in geothermal systems, and (iii) determine which “universal” archaeal 16S rRNA gene primers are interrupted by the presence of intron sequences.

## Results and discussion

**Fig. 1:**
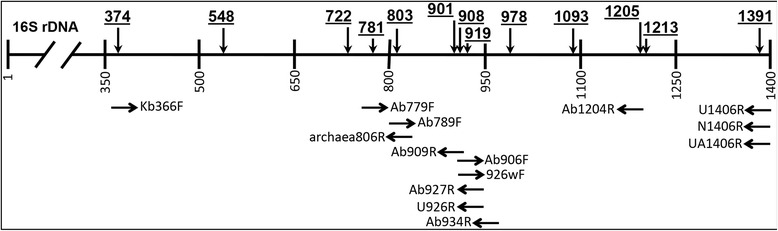
Position of introns identified in 16S rRNA genes within the *Archaea*. Vertical arrows indicate loci with positions underlined (*E. coli* 16S rRNA numbering). Horizontal arrows indicate “universal” archaeal primers interrupted by the presence of intron sequences (arrows not to scale)

**Fig. 2:**
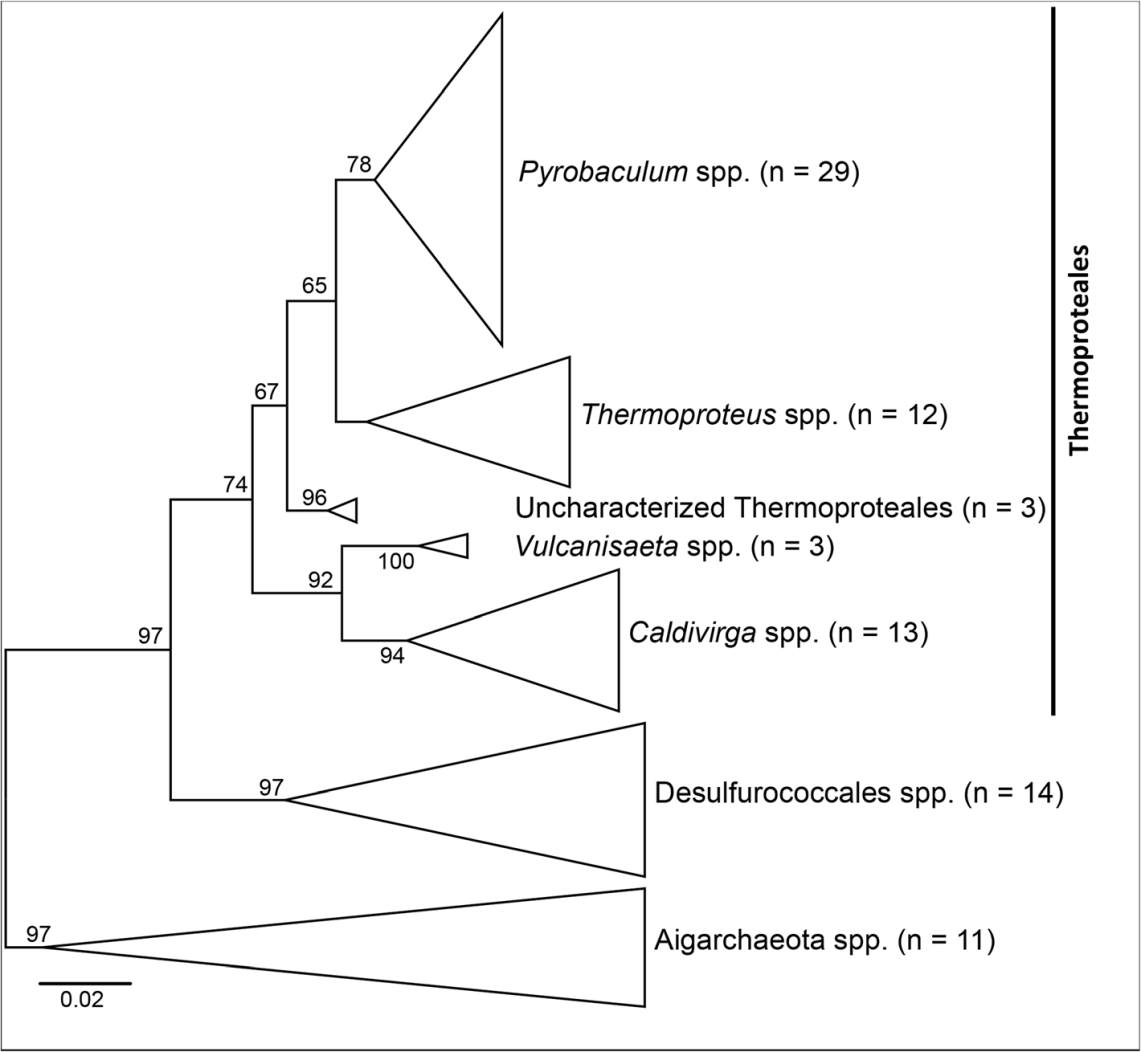
Phylogenetic tree of 16S rRNA genes that contain intron sequences. Intron sequences were not included in the alignments. The tree was constructed with Neighbor-Joining methods and bootstrap values were determined by resampling 1000 trees

Intron sequences were confined to 13 loci across the 16S rRNA gene (Fig. 1, Table 1). The number of introns identified per locus ranged from 2 (locus 722) to 41 introns (locus 781). Phylogenetic analysis of host 16S rRNA genes revealed that the large majority of introns (>90 %) were observed within the order Thermoproteales, and less in the Desulfurococcales (Crenarchaeota) and Aigarchaeaota (Fig. 2). *Pyrobaculum* spp. contained the majority of known introns and these are found at diverse loci across the 16S rRNA gene (loci 374, 548, 781, 907, 908, 919, 1093, 1205, 1213, 1391). Other genera in the Thermoproteales also contain intron sequences at many of the same loci: *Thermoproteus* (loci 548, 722, 1093, 1205, 1213, 1391), *Caldivirga* (loci 374, 781, 901, 908, 919), and *Vulcanisaeta* (locus 1391). Desulfurococcales-like introns were identified in six loci (548, 901, 908, 919, 1093, 1205).

**Table 1:** Intron insertion loci identified in archaeal 16S rRNA genes

Representative Taxa	Locus^a^	#CDS^b,c^	#HP^2^	#PRU^b,c^	Interrupted “universal” primer(s)
*Pyrobaculum*, *Caldivirga*	374	5 (5,0)	10	-	Kb366F
*Pyrobaculum*, *Thermoproteus*, *Staphylothermus*, Desulfurococcales^d^	548	2 (0,2)	13	-	-none-
*Caldiarchaeum*	722	-	-	2	-none-
*Pyrobaculum*, *Thermoproteus*, *Vulcanisaeta*, Thermoproteales^d^, *Caldivirga*, *Caldiarchaeum*	781	14 (2,4)	-	28	Ab779F
Desulfurococcales	803	-	-	3	Ab789F, Arc806R
*Pyrobaculum*, *Caldivirga*, Desulfurococcales	901	-	8	-	Ab909R
*Pyrobaculum*, *Caldivirga*, *Aeropyrum*, *Caldiarchaeum*	908	4 (1,3)	-	6	Ab909R, Ab906F, 926wF, U926R, Ab927R
*Pyrobaculum*, *Thermoproteus*, *Caldivirga*, *Vulcanisaeta*, *Caldiarchaeum*, Desulfuroccocales	919	6 (2,3)	-	27 (1,0)	Ab906F, 926wF, U926R, Ab927R, A934R
*Caldiarchaeum*	978	-	9	-	-none-
*Pyrobaculum*, *Thermoproteus*, Desulfurococcales	1093	15 (4,11)	7	9 (2,1)	-none-
*Pyrobaculum*, *Thermoproteus*, Desulfurococcales	1205	2 (0,2)	17	-	-none-
*Pyrobaculum*, *Thermoproteus*	1213	18 (4,14)	-	2	-none-
*Pyrobaculum*, *Thermoproteus*, *Vulcanisaeta*	1391	15 (7,2)	-	5	UA1406R, N1406R, U1406R

^a^Intron insertion location in 16S rRNA gene (*E. coli* numbering, nucleotide position directly before insertion), ^b^ *CDS* Homing endonuclease Coding Sequence, *HP* small (<50 nt) RNA hairpin-forming introns, *PRU* Partial, Remnant, or Uncharacterized introns, ^c^Numbers in parenthesis refer to the number of LAGLI-DADG motifs (1 or 2, respectively), ^d^Specifies an uncharacterized phyla within the Desulfurococcales and Thermoproteales, respectively

Most (9/13) intron regions contained two or more predicted homing endonuclease genes, which code for either one or two of the canonical LAGLI-DADG motifs (Table 1). Short (<50 nt), hairpin-forming introns were identified in 7 intron loci, and intron loci 901, 978, and 1205 only contained short hairpins. Partial, remnant, or undetermined (PRU) intron sequences were identified in eight of the 13 loci. The activity of *P. oguniense* 16S rRNA homing endonuclease (Pog.S1213) promoted the homing of its own intron and guaranteed the co-conversion of both the downstream hairpin-forming intron (Pog.S1205) and the intervening eight nucleotides by cleaving at the intron^−^ locus 1205 [38]. This co-conversion homing mechanism may be applicable to other 16S rRNA gene loci, specifically introns at loci 901 (HP-only) and 908 (homing endonuclease encoding), which are separated by seven nucleotides.

### Secondary structures of intron sequences

**Fig. 3:**
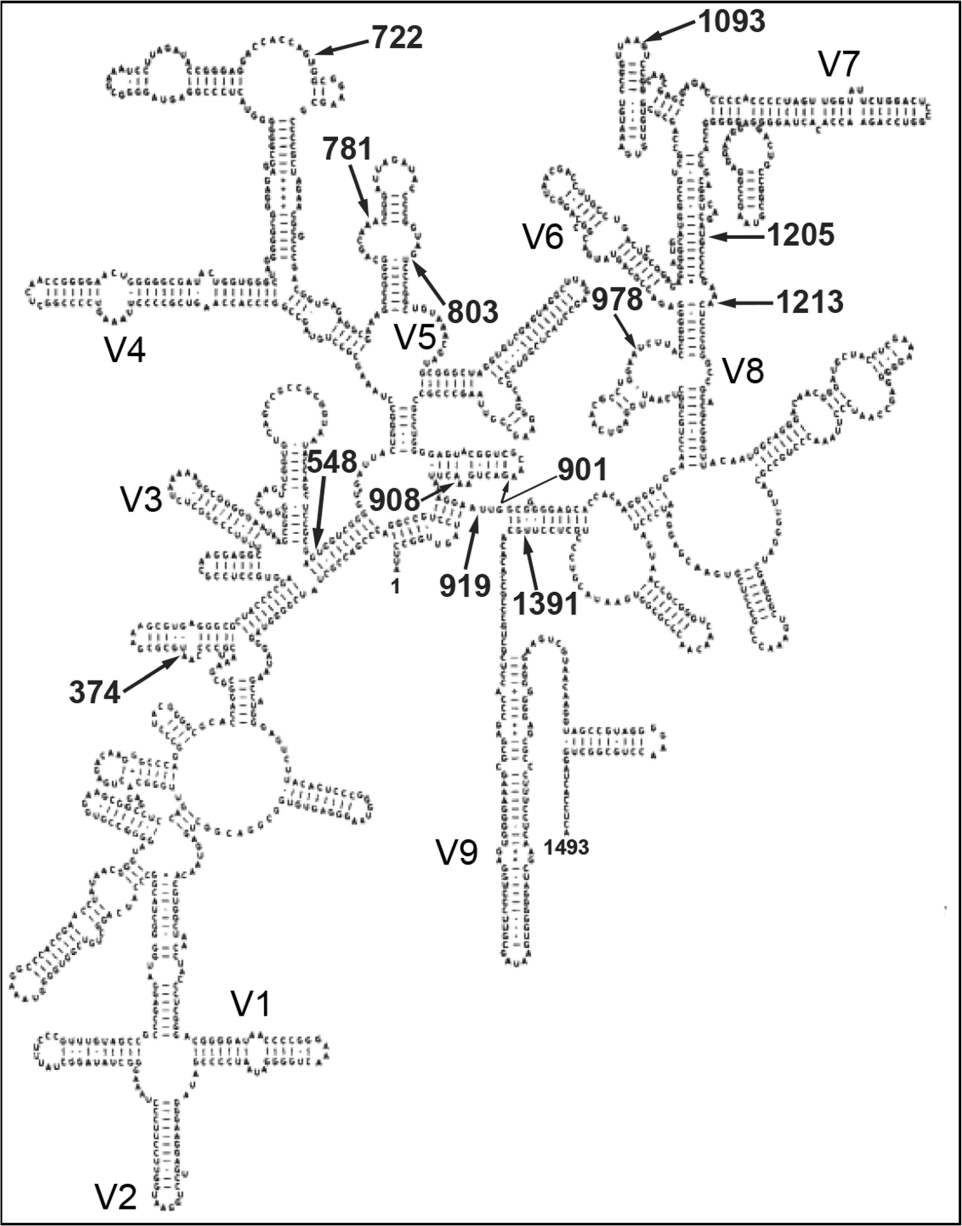
The location (*E. coli* numbering) of intron sequences (identified in the current study) within the transcribed secondary structure of the 16S rRNA gene. Variable regions (V1 - V9) are shown for reference

Analysis of the intron insertion loci in the modeled secondary structure of an archaeal 16S rRNA molecule [39] revealed that nearly all of the intron loci (loci 374, 722, 781, 803, 901, 908, 919, 978, 1093, 1213, 1391) were either located in a bulge motif or in a helix structure very near a bulge motif (548, 1205; Fig. 3). These locations may provide the necessary flexibility in secondary structure for the insertion of intron sequences, which are often > 700 nt. There are many other bulge motifs in the secondary structure of the 16S rRNA molecule that do not contain intron sequences. This might be attributed to secondary or tertiary structure restrictions (e.g., steric hindrance), sequence specificity of insertion, or an inability to detect introns at these locations in the environment. Alternatively, these loci may not provide sufficient access to the tRNA splicing endoribonuclease. All V-regions (V1 - V9; [40]) of the 16S rRNA gene were free of introns, indicating that many introns are confined to the most highly-conserved loci in the 16S rRNA gene. Intron loci 374 and 803 flank variable regions V4 and V8, respectively, and could interrupt primers designed around these regions (see below).

**Fig. 4:**
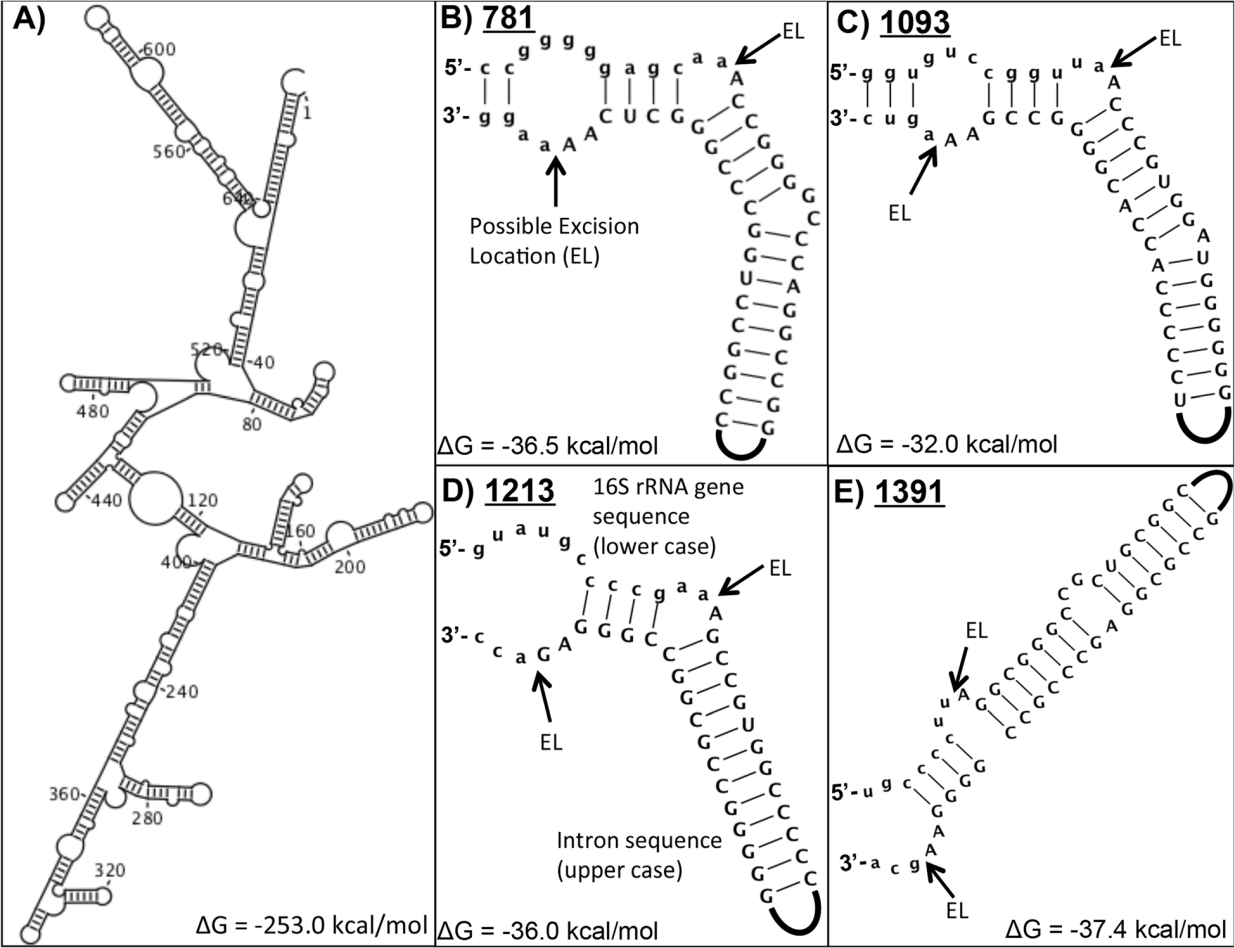
Predicted secondary structures of 16S rRNA gene introns. **a** Predicted secondary structure of the *Pyrobaculum yellowstonensis* strain WP30 16S rRNA gene intron at locus 1391, illustrating the highly-structured nature of transcribed intron sequences. Numbers denote nucleotide position along the intron sequence. **b**-**e** Predicted secondary structures based on consensus sequences (weblogo) of four different 16S rRNA gene introns. Lower and upper case nucleotides denote 16S rRNA gene sequence and intron sequence, respectively. Arrows indicate hypothesized excision locations (EL) within the bulge-helix-bulge motif

Transcribed intron sequences also had predictable, thermodynamically favorable secondary structures (e.g., Fig. 4a), similar to observations of 23S rRNA gene introns [25]. Intron sequences sharing high nucleotide identity within the same insertion locus maintained similar secondary structure (data not shown). However, the predicted secondary structures were not generally conserved within insertion loci. The predicted folding of each transcribed intron is thermodynamically favorable (at 37 °C; Fig. 4), but considering the predominance of introns in high-temperature habitats, the themostability of secondary structures may play a yet uncharacterized role in intron distribution and propagation. The average G + C content of the intron sequences was 57 ± 11 %, indicating that some thermostability could be attributed to strong G + C bonding; however, this value is still lower than the average % G + C content of the host 16S rRNA genes (67 ± 2 %).

Comparison of intron sequences within the same loci identified highly-conserved nucleotide residues near the intron-exon junctions, which represent conserved intron cores [14, 25]. These cores form a bulge-helix-bulge (BHB) motif (Fig. 4 b-e) post-transcription that is very similar to the characterized BHB motifs of intron-containing archaeal tRNAs [41] and 23S rRNAs [23], both of which are excised by the same splicing endoribonuclease [23, 42–44]. The order Thermoproteales, and especially the genus *Pyrobaculum*, contain the majority of intron-containing tRNAs and 23S rRNA gene introns in the domain *Archaea*. The flanking 16S rRNA gene sequences of each intron locus were also highly conserved across entries in the domain *Archaea* (also one of the reasons “universal” primers have been designed around these loci). The nucleotide sequences comprising the DNA insertion sites and the BHB motif vary among loci and it is unclear how sequence specificity of the homing endoribonuclease or the splicing ligase contributes to intron propagation and distribution. Several intron loci were identified in both the Crenarchaeota and Aigarchaeota (e.g., loci 908, 919, 1093, and 1205), which suggests that these loci have been sites of intron insertions since the divergence of these two phyla. Based on extant insertion loci, intron sequences radiated throughout the Thermoproteales, specifically in the *Pyrobaculum* and *Thermoproteus* spp.

### Phylogenetic analysis of 16S rRNA gene introns

**Fig. 5:**
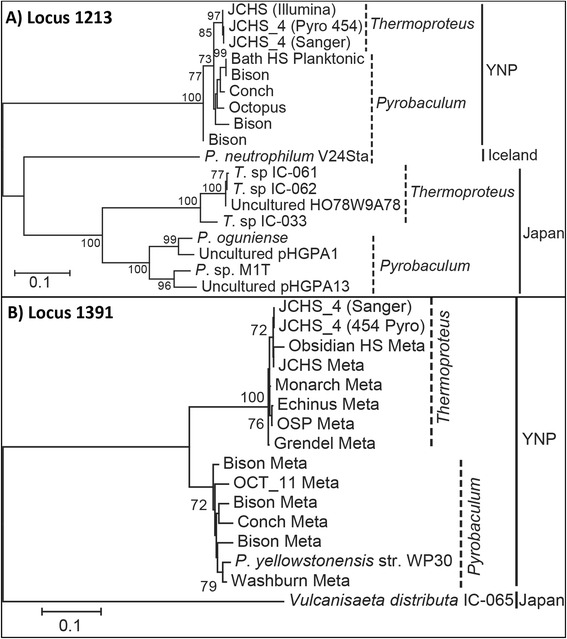
Phylogenetic analysis of intron sequences (homing endonuclease encoding) at loci 1213 (**a**) and 1392 (**b**). Unrooted trees were constructed with Neighbor-Joining methods and bootstrap values were determined by resampling 1000 trees

Intron sequences within the same loci were successfully aligned at the nucleotide level and subsequent phylogenetic analysis revealed both intra-locus and geographic separation (Fig. 5). Intron sequences within loci tended to clade with other introns from similar geographic locations, rather than with related genera. For example, the Thermoproteales-like homing endonuclease encoding genes at loci 781, 1093, and 1213 (shown in Fig. 5a) formed clades corresponding to sequences from Japan, YNP, Kamchatka, and/or Iceland and not by genus, although Kamchatka and Iceland were only represented by solitary isolates *Pyrobaculum* sp. 1860 [12] and *P. neutrophilum* [45], respectively. Intron sequences grouped first by geographic location (Fig. 5); for example, intron sequences from Japan grouped together compared to entries from YNP (i.e., loci 781 and 1213). Locus 1391 contained several sequences from YNP, including a novel intron identified in the YNP isolate *P. yellowstonensis* strain WP30 [46] that is highly related to intron sequences obtained from the Bison Pool metagenome (Fig. 5b). The first, and only, previously described intron at this location was a *Vulcanisaeta distributa* IC-065 clone from Japan [18]; however this sequence is significantly divergent from the YNP entries. These entries clade based on the phylogeny of the host 16S rRNA gene (i.e. *Pyrobaculum* and *Thermoproteus*). Introns found at different 16S rRNA gene loci were too different for phylogenetic comparison (<20 % nt/aa identity).

The phylogenetic distribution of intron sequences by geographic location is consistent with the hypothesis that once a homing endonuclease becomes fixed in a genome, selection pressure for the gene significantly decreases resulting in degeneration (mutation) and enforcing the requirement of frequent horizontal transmissions between populations to maintain intron propagation [34]. Populations related to the genus *Pyrobaculum* contain the most abundant 16S rRNA introns in the order Thermoproteales and although they predominate in high-temperature, near-neutral to alkaline (pH > 6) geothermal systems, populations of the other Thermoproteales genera (*Caldivirga*, *Vulcanisaeta* and *Thermoproteus*) are found with *Pyrobaculum* in > 65 °C, pH 5 - 7 hot springs [47, 48]. Therefore, the optimal conditions for intron transmission among the Thermoproteales may lie within this very defined temperature and pH range. Considering that rRNA introns are likely ancient [49], these environmental conditions may reflect constraints on the origin of RNA gene introns. Introns may be perpetuated in extant Thermoproteales species and result in genetic variation, analogous to the extensive mobile elements identified in some Sulfolobales genomes [50]. Very little is known about the dispersal of thermophilic archaea, however, many intron sequences were highly similar among YNP habitats (e.g., locus 1391). Additional archaeal 16S rRNA introns obtained from other geothermal systems will help resolve observed patterns of intron distribution and diversity.

### “Universal” 16S rRNA gene primers

Primer sequences designed for archaeal 16S rRNA genes (n = 51) were manually aligned to intron-containing 16S rRNA gene sequences to determine which primers were interrupted by, or spanned intron sequences (Fig. 1). Fourteen primers spanned at least one intron insertion locus, including five primers that spanned two insertion loci (Ab909R, Ab906F, 926wF, U926R, Ab927R) and are located near the center of the 16S rRNA gene. “Universal” primers designed to anneal near the middle of the 16S rRNA gene have the greatest potential of being interrupted by an intron sequence. The remaining archaeal primers evaluated here did not directly overlap with currently known introns; however, depending on the chosen complementary primer for PCR, the resulting amplicons may contain complete or partial intervening intron sequences. These non-16S rRNA gene fragments are then often included in long-fragment 16S rRNA gene sequences, and have a high probability of being misinterpreted (either by human or computer) as non-specific amplification, chimeras, sequencing error, and/or assembly error, which may result in their exclusion from public databases. The failure to detect intron-containing 16S rRNA genes in thermal habitats using many universal primers would potentially underestimate several abundant Thermoproteales organisms. Consequently, specific efforts to quantify members of the Thermoproteales using short or long-fragment sequencing technologies should be aware of intron-containing 16S rRNA genes, and avoid primer sets that are centered on common sites of intron sequence, or intron insertion loci.

## Conclusions

Intron sequences were confined to 13 loci across the 16S rRNA gene and were most abundant in the order Thermoproteales (phylum Crenarchaeota). All transcribed introns form predictable secondary structure including the characteristic bulge-helix-bulge motif. Many intron sequences encoded homing endonucleases, although other introns were short and/or non-coding sequences. Phylogenetic analysis revealed that intron sequences grouped together by geographic location and then by host taxa. The presence of introns in highly conserved regions of 16S rRNA introns directly interferes with use of “universal” primers often used in environmental gene surveys.

## Methods

Intron sequences were identified by querying (blastn/blastx) the NCBI nr and the DOE-Joint Genome Institute (DOE-JGI) Integrated Microbial Genomes (with Microbiome Samples (IMG/M) databases) with previously identified 16S gene introns (Additional file 1: Table S1). Approximately 100 16S rRNA genes were identified that contained over 180 intron sequences (Additional file 2: Table S2) resulting in a total dataset of ~230 intron sequences (in ~115 16S rRNA genes) distributed at 13 different loci across the entire length of the 16S rRNA gene (Fig. 1, Table 1). Homing endonucleases were identified and secondary structures were predicted using CLC Main Workbench (v6.9.1; Qiagen). Longer intron sequences (> ~100 nt) were translated to amino acid sequence (if possible) and searched against the Pfam database (v. 27.0; [51]) to identify LAGLI-DADG motif(s) that are indicative of homing endonucleases [33]. Intron sequences were grouped into the following categories: (i) introns encoding a homing endonuclease, (ii) introns without an obvious open reading frame (remnant), (iii) introns forming small (< ~ 50 nt) predictable hairpin structures that maintain the bulge-helix-bulge motif, or (iv) partial, truncated, or uncharacterized intron sequences.

Sequence alignments were performed (manually and/or with ClustalW) before phylogenetic analysis and/or Weblogo3 analysis [52]. Phylogenetic trees of 16S rRNA genes and intron sequences were constructed by employing Neighbor-Joining or Maximum likelihood methods (Mega 5.2.2; [53]). Over 50 “universal” archaeal primers were manually aligned to 16S rRNA genes to identify those interrupted by, or that spanned intron loci (Additional file 3: Table S3).

## Reviewers’ comments

### Reviewer 1: Dr. Eugene Koonin

**Report form:** Jay and Inskeep report the distribution of Group I introns in 16S RNA genes from hyperthermophilic archaea, and in particular Thermoproteles, where these introns are most common. The analysis is carefully performed, and there is an important conclusion on the evolution of self-splicing introns, namely that they group by geographic location, i.e. spread primarily horizontally. Also, this analysis is of practical value because the authors carefully assess the effect of introns on the use of universal primers.

Authors’ response: We thank the reviewer for these comments.

**Quality of written English:** Acceptable.

### Reviewer 2: Dr. W. Ford Doolittle

**Report form:** This is a perfectly fine and straightforward report and summary of the distribution of introns in the rRNA genes of members of the Thermoproteales. It will provide a basis for future experimental and comparative studies, and raises some interesting questions, such as the means by which introns are transferred between different lineages in a given environment and the structural adaptations of the intron RNA to high temperature, but does not attempt to answer them. It also points out how intron presence might result in failure to amplify specific regions of rRNA genes by PCR. Members of Thermoproteales might thus be underestimated in environmental sampling, and one wonders whether there are abundant major groups that go undetected because they always have such introns. Indeed, is there anything to keep introns from becoming colonized by genes essential for cellular survival, so that pieces of the rRNA gene become permanently separated, and the cells that bear them become undetectable with standard molecular methods?

Authors response: We thank the reviewer for these comments, and for the very interesting question regarding possible ‘colonization’ of introns by genes coding for essential function, and subsequent separation of rRNA gene fragments (i.e., within the genome). The question regarding intron colonization by other genes is difficult to answer due to the complexities of intron propagation. As we currently understand 16S rRNA gene introns in the Thermoproteales, a homing endonuclease (gene or transcript) is required for intron insertion into a functional rRNA. Consequently, any disruption to the homing endonuclease would not allow for insertion and propagation of the intron sequence. Any cell that may bear intron fragments by definition will still have a functioning rRNA somewhere in the genome and therefore would be detectable with standard methods.

**Quality of written English:** Acceptable.

## Additional files

Additional file 1: Table S1.
*Archaea* with known 16S rRNA gene introns. Additional file 2: Table S2. Environmental metadata of sites in Yellowstone National Park where 16S rRNA gene introns were identified. Additional file 3: Table S3. “Universal” archaeal 16S rRNA gene primers interrupted by introns common in members of the Thermoproteales (phylum Crenarchaeota).

## Abbreviations

BHB: Bulge-helix-bulge
YNP: Yellowstone National Park
RtcB: tRNA splicing ligase
