# Supplementary material for: The distribution, diversity, and importance of 16S rRNA gene introns in the order Thermoproteales
Source: Biol Direct. 2015 Jul 9;10:35. doi: 10.1186/s13062-015-0065-6 (PMC4496867; doi:10.1186/s13062-015-0065-6)
Supplement: Additional file 1: Table S1. — Archaea with known 16S rRNA gene introns. [file 13062_2015_65_MOESM1_ESM.pdf]

Table S1. *Archaea* with known 16S rRNA gene introns.

Reference	Location	Temp (°C)	pH	16S rDNA Intron	
				**Reg.**	**Type**
Pyrobaculum aerophilum	Italy	100	6.0	374	CDS (1)
P. arsenaticum	Italy			374	CDS (1)
P. sp. 1860	Kamchatka	84	6.8	374	CDS (1)
				1093	CDS (2)
P. sp. M1T	Japan			374	CDS (1)
				781	CDS (2)
				1093	CDS (2)
				1205	HP
				1213	CDS (2)
Thermoproteus sp. IC-033	Japan	95		548	CDS (2)
				781	CDS (1)
				1093	CDS (1)
				1205	HP
				1213	CDS (2)
Staphylothermus marinus		85	6.5	548	HP
P. sp. M0H	Japan			781	CDS (2)
P. sp pHGPA13 (clone)	Japan	96		781	unk
				901	HP
				908	CDS (2)
				1205	HP
				1213	CDS (1)
P. sp pHGPA1 (clone)	Japan	96		781	CDS (1)
				1205	HP
				1213	CDS (1)
T. sp. IC-061	Japan	95	3.1	781	CDS (2)
				1205	HP
				1213	CDS (1)
Caldivirga maquilingensis	Japan	85	3.9	901	HP
				908	unk
Aeropyrum pernix	Japan	92.5	7.0	908	CDS (2)
Caldiarchaeum subterraneum	Japan	69	5.1	908	CDS (2)
				919	unk
P. yellowstonensis WP30	YNP	75	6.0	919	CDS (2)
				1093	HP
				1391	CDS (1)
P. neutrophilum	Iceland	85	6.5	1205	HP
				1213	CDS (2)
P. sp. IC-062	Japan	95	3.1	1205	HP
				1213	CDS (2)
P. oguniense	Japan	92	6.6	1205	HP
				1213	CDS (2)
Vulcanisaeta distributa IC-065	Japan	90	2.2	1391	unk
