# Supplementary material for: The distribution, diversity, and importance of 16S rRNA gene introns in the order Thermoproteales
Source: Biol Direct. 2015 Jul 9;10:35. doi: 10.1186/s13062-015-0065-6 (PMC4496867; doi:10.1186/s13062-015-0065-6)
Supplement: Additional file 2: Table S2. — Environmental metadata of sites in Yellowstone National Park where 16S rRNA gene introns were identified. [file 13062_2015_65_MOESM2_ESM.pdf]

Table S2. Environmental metadata of sites in Yellowstone National Park where 16S rRNA gene introns were identified.

Environmental Sample	T(°C)	pH	Year Sampled	Method[1]	# 16S w/ introns	Insertion Loci[2]
**Cistern Spring (YNP)**						
CIS_19	78	4.4	2007	SM	1	919 (1), 1391 (1)
CisS	76	4.4	2011	IM	3	781 (1), 919 (1), 1093 (1), 1205 (1)
**Joseph's Coat HS (YNP)**						
JCHS	89.8	6.1	2004	C	10	781 (1), 919 (2), 1093 (7), 1391 (1)
Ystone1	78-82	6.1	2005	SM	1	1093 (1)
JCHS_4	76-80	6.1	2007	SM	3	374 (1), 781 (1), 919 (1), 1093 (2), 1205 (1), 1213 (1)
JCHS454	76-80	6.1	2007	454M	5	374 (1), 781 (2), 919 (2), 1093 (3), 1205 (1), 1213 (2), 1391 (1)
JC3ASed	76-80	6.1	2011	IM	5	374 (1), 781 (2), 919 (2), 1213 (1), 1391 (1)
**Monarch Geyser (YNP)**						
MG_3	80		2007	SM+454M	1	919 (1)
Monarch	75-85		2011	IM	5	374 (2), 781 (1), 919 (1), 1093 (1), 1205 (1), 1391 (1)
**Octopus Spring (YNP)**						
OCT_11	78-82	8	2007	SM	1	781 (1), 919 (1), 1391 (1)
OCT	84	7.8	2011	IM	1	1205 (1), 1213 (1)
OCTB	82-84	8.7	2012	IM	2	781 (1), 919 (1)
**Conch Spring (YNP)**						
ConS	84-85	8	2011	IM	1	781 (1)
ConchC	84-85	8	2012	IM	2	781 (1), 1391 (1)
**One Hundred Spring Plain (YNP)**						
OSPB	72	3.3	2010	454M	1	374 (1), 781 (1), 919 (1)
OSP	74-76	3.5	2011	IM	4	374 (2), 781 (3), 919 (2), 1391 (1)
**Grendel Spring (YNP)**	80	3.4	2011	IM	3	374 (1), 781 (2), 919 (1), 1391 (1)
**Echinus Geyser (YNP)**	68	3.4	2011	IM	4	374 (1), 781 (3), 919 (1), 1391 (1)
**Joseph's Coat HS 2E (YNP)**	78-80	2.2	2011	IM	2	374 (1), 781 (1), 919 (2)
**Washburn Spring (YNP)**						
WS_18	74	6.4	2007	SM	1	919 (1)
Washburn	76	6.4	2011	IM	2	781 (1), 1391 (1)
**Bechler Spring (YNP)**						
BCH_13	82	7.8	2007	M	1	781 (1)
**Bath Hot Spring (YNP)**						
BHS-Planktonic	86	9.3	2005	M	4	781 (2), 1093 (2), 1213 (1), 1391 (2)

**Bison Pool (YNP)**						
BP1	82.6	8.1	2001	C	3	548 (3), 901 (2), 919 (2), 1093 (1), 1205 (1)
BP2A	78.1	7.7	2005-06	C	2	548 (2)
BP2B	79.6	7.7	2005-06	C	1	548 (1)
BP4	65.6	8.0	2005-06	C	1	908 (1), 978 (1)
BPA	92.1	7.3	2005	SM	4	919 (1), 1093 (3), 1205 (2), 1213 (1), 1391 (2)
BPB	78.6	7.7	2005	SM	8	548 (2), 781 (1), 803 (1), 901 (2), 919 (3), 1093 (2), 1205 (1), 1213 (3), 1391 (2)
**Mound Spring (YNP)**						
MS1	87.5	8.8	2005-06	C	1	548 (1), 803 (1)
MS2	74.1	9.2	2005-06	C	5	548 (2), 781 (3), 901 (1), 919 (1)
**Queen's Laundry (YNP)**						
QL1	75.7	7.7	2005-06	C	2	548 (1), 803 (1), 908 (1), 978 (1)
QL2	72.8	7.9	2005-06	C	6	908 (3), 978 (6)
**Obsidian HS (YNP)**	60?	6.4	2005-06	SM	2	1093 (1), 1391 (1)
**Subterranean Gold Mine (Japan)**	69	5.1	2001-03	C	3	722 (1), 908 (1)
**Yunohama (Japan)**	57	8.1	2009	C	1	722 (1), 908 (1)
**Ohwakudani, Hakone (Japan)**	78	3.5	2009	C	3	781 (2), 1205 (1), 1213 (1)

[1]Method and sequencing technology used to generate sequences (C = clone, SM = Sanger Metagenome, 454M = 454-Pyrosequencing Metagenome, IM = Illumina Metagenome)
[2]Intron position (*E. coli* numbering) and total number of introns at that position included.
