# Supplementary material for: The distribution, diversity, and importance of 16S rRNA gene introns in the order Thermoproteales
Source: Biol Direct. 2015 Jul 9;10:35. doi: 10.1186/s13062-015-0065-6 (PMC4496867; doi:10.1186/s13062-015-0065-6)
Supplement: Additional file 3: Table S3. — “Universal” archaeal 16S rRNA gene primers interrupted by introns common in members of the Thermoproteales (phylum Crenarchaeota). [file 13062_2015_65_MOESM3_ESM.pdf]

Table S3. "Universal" archaeal 16S rRNA gene primers interrupted by introns common in members of the Thermoproteales (phylum Crenarchaeota).

Primer	Sequence (5'-3')	Locus
Kb366F	CTCCGCAATRCGCGMAAG	374
Ab779F	GCRAASSGGATTAGATACCC	781
Ab789F	TAGATACCCSSGTAGTCC	803
archaea806R	GGACTACVSGGGTATCTAAT	803
Ab909R	TTTCAGYCTTGCGRCCGTAC	901, 908
Ab906F	GAAACTTAAAKGAATTG	908, 919
926wF	GAAACTYAAAKGAATTGRCGG	908, 919
U926R	CCGTCAATTCCTTTRAGTTT	908, 919
Ab927R	CCCGCCAATTCCTTTAAGTTTC	908, 919
A934R	GTGCTCCCCCGCCAATTCCT	919
UA1204R	TTMGGGGCATRCNKACCT	1205
UA1406R	ACGGGCGGTGWGTRCAA	1391
N1406R	ACGGGCGGTGAGTGCAA	1391
U1406R	GACGGGCGGTGTGTRCA	1391
